# Optimization of Image Quality and Organ Absorbed Dose for Pediatric Chest X-Ray Examination: In-House Developed Chest Phantom Study

**DOI:** 10.1155/2022/3482458

**Published:** 2022-04-16

**Authors:** Thanyawee Pengpan, Natch Rattanarungruangchai, Juthathip Dechjaithat, Phawinee Panthim, Puntarika Siricharuwong, Ausanai Prapan

**Affiliations:** ^1^Department of Radiological Technology, Faculty of Allied Health Sciences, Naresuan University, Phitsanulok 65000, Thailand; ^2^Department of Radiation Dose Measurement and Assessment, Nuclear Technology Service Center, Thailand Institute of Nuclear Technology (Public Organization), Bangkok, 26120, Thailand

## Abstract

**Purpose:**

This study aimed to identify proper exposure techniques to maintain optimal diagnostic image quality with minimum radiation dose for anteroposterior chest X-ray projection in pediatric patients.

**Methods:**

Briefly, an in-house developed pediatric chest phantom was constructed. Next, nanodot OSLDs were used for organ absorbed dose measurement and placed in the lung area, and the phantom was exposed to various exposure techniques (ranging from 50 to 70 kVp with 1.6, 2, and 2.5 mAs). After that, the phantom was used to assess image quality parameters, including SNR and CNR. Two radiologists assessed the subjective image quality using a visual grading analysis (VGA) technique. Finally, the figure of merit (FOM) was analyzed.

**Results:**

The developed phantom was constructed successfully and could be useful for dose measurement and image quality assessment. The absorbed dose varied from 0.009 to 0.031 mGy for the range of exposure techniques used. SNR and CNR showed a gradually increasing trend, while kVp and mAs values were increased. The highest kVp (70 kVp) produced the highest SNR and CNR, exhibiting a significant difference compared with 50 and 60 kVp (*P* < 0.05). The overall VGA score was 3.2 ± 0.3, and the low kVp technique demonstrated better image quality compared with the reference image.

**Conclusion:**

The optimized exposure technique was identified as 60 kV and 2.5 mAs, indicating the highest FOM score. This work revealed practicable techniques that could be implemented into clinical practice for performing pediatric chest radiography.

## 1. Introduction

Chest radiography plays a vital role in the performance of image examination on pediatric patients. Further, it is essential for obtaining a rapid diagnosis in pediatric clinical practice to plan and monitor treatment. Ionizing radiation is necessary to generate the chest radiograph for providing diagnostic information, and its use must be considered regarding the risk of radiation-related adverse effects and consequences. There is a state of heightened risk among children and neonates due to being more sensitive to ionizing radiation than adults [1]. Moreover, their longer life expectancy indicates they have a higher likelihood for repeated exposure and the appearance of potentially harmful effects of radiation compared with adults [2, 3]. Previous studies pointed out that children face approximately ten times higher risk than adults for cancer induction [4].

Regarding the rapidly increasing use of ionizing radiation in diagnostic radiography, radiographers must be cognizant about patient dose and the factors that affect radiation dose to avoid subjecting patients to unnecessary increases in radiation. Particularly, radiographers should have familiarity and understanding of clinical examinations to preserve the low risk of radiation without compromising the diagnostic accuracy of pediatric radiographs [5, 6]. Moreover, several studies have reported that patients undergoing diagnostic X-ray examinations tend to have limited knowledge about medical radiation and its associated risks [7]. Therefore, it is necessary to optimize and estimate the radiation dose with the goal of alleviating induced biological effects, especially in pediatric patients.

Optimization is considered by the implementation of the ALARA principle, indicating that radiation levels are maintained as low as reasonably achievable (ALARA). Optimization also represents the recognition of the required level of radiographic image for diagnostic purposes and determining which radiographic technique/exposure achieves that level of image quality while delivering the least possible amount of radiation [8]. Previous studies demonstrated that increasing the benefits to patients' health while decreasing the risk of biological effects was implied under the optimization concept, which points out the need to determine the most appropriate radiation dose for patients.

Considered a chest radiography procedure, many pediatric patients do not cooperate during an X-ray examination and are restless. Therefore, phantoms are commonly used instead of pediatric patients for the investigation of image quality and radiation dose. Phantoms are generally constructed from tissue-equivalent materials and should be more representative of realistic physical characteristics, including the simulation of human anatomy and attenuation characteristics [9–12].

Our study aimed to investigate the organ absorbed dose and image quality optimization on the anteroposterior (AP) projection of pediatric chest X-ray examination by using a novel constructed in-house chest phantom through the variation of exposure technique setting. The proper selection of exposure techniques and related settings produces a diagnostically acceptable radiograph with a minimal patient radiation dose.

## 2. Materials and Methods

### 2.1. Pediatric Chest Phantom

#### 2.1.1. Design, Material Selection, and CT Validation

The phantom was designed to simulate the average chest size of the 1-2 years old, and thorax dimension data was obtained from the standard body size of Thai children (1–2 years old) [13]. The phantom was invented using materials designed to mimic human tissue. The tissue equivalent substitutes materials were chosen and developed a similar response to the physical properties of human tissue, including density. Ideally, our phantom should have a similar human anatomical shape and matching dimensions and be easily constructed from the correct tissue substitute material.

For selecting an appropriate tissue substitute, the tissue-equivalent materials were chosen as follows: (a) soft tissue-equivalent substitute (STS): a polyester resin (POLYLINE PC600-S Casting Resin, purchased from Rungroj Fiberglass Ltd., Bangkok, Thailand) was chosen and designed to nearly matching the density of human soft tissue. Moreover, the polyester resin was used in designing because it is readily available, easy to work with at room temperature, and durable and is considered a low-cost material [14]. (b) *Bone Tissue-Equivalent Substitute (BTS).* The mixture of the polyester resin with 40% CaCO_3_ was used for the construction of bone tissue in the chest phantom, as previously described. [15] (c) *Lung Tissue-Equivalent Substitute (LTS).* The polyurethane foams were used as a fully inspired human lung. The density of organs and the tissue substitute were demonstrated in Table 1.

Moreover, the computed tomography (CT) validation of the tissue substitute materials should be carried out. The conformity of the CT numbers in the tissue substitute was investigated. At first, the samples of STS, BTS, and LTS were prepared with the square shape of dimensions 10 × 10 × 1 cm. After that, a CT scan (Philips Ingenuity core 64 slice CT, Philips Medical Systems, Cleveland, USA) was performed to obtain CT images in DICOM format under the data acquisition for pediatric thorax 0–10 kg protocol, including 100 kV, 100 mAs, 0.5 sec rotation time, 3 mm slice thickness, and 1.5 mm reconstruction interval. To determine the CT number of the sample, the region of interest (ROI) with dimensions of 100 mm2 was selected in the axial view of CT images of each slice, and then, the CT number (HU) of each slice was measured. Table 1 also shows a comparison of the CT number of our tissue-equivalent substitute used and the standard reference data, which have been reported in much previous research [16–19].

#### 2.1.2. Construction of Pediatric Chest Phantom

The pediatric chest phantom assembly process was conducted using the method described previously [15]. The method involved several steps as follows: briefly, a mold-like pediatric thorax shape was made from the fiberglass according to the data of the pediatric standard size. The bony parts of the phantom (ribs, sternum, and vertebrae segments) were assumed and produced similar to the anatomical configuration in the silicone mold by using the BTS as mentioned in Section 2.1.1. Next, all BTS were placed symmetrically and accurately in a position similar to the anatomical structure in the fiberglass mold. After that, the polyester resin-based STS materials were prepared and poured carefully into the thorax mold, milling out the appropriate voids for inserting the LTS materials. To fabricate the lung inserts, separate molds were produced using polyurethane foam-based LTS materials, and then, the wax was used to coat the surface for stability and smoothness.

### 2.2. Image Equipment and Setting

The Hyundai 500 mA Stationary X-ray Machine (Model IMAGE-X50, Hyun Dai Medical X-ray Co., Ltd., Yongdeungpo-Gu, Seoul, Korea) was used. All radiographic exposure was performed with a broad focal spot of 1.0 mm, 24 × 30 cm collimation, 100 cm SID, and no grid. The Computed Radiography (CR) System (DirectView vita CR system, Carestream Health, Inc. Rochester, New York, USA) was used together with an imaging plate for this experimental setup. Before starting the study, quality control testing of the X-ray machine was performed and results fell within the acceptable tolerances limit.

Fifteen radiographs were created in this study. Images were produced by using the various exposure settings as follows: kVp settings were 50, 55, 60, 65, and 70. For each kVp, three mAs settings were used: 1.6, 2, and 2.5 mAs, respectively.

### 2.3. Absorbed Dose Measurement

The nanodots OSLDs have been extensively used as a tool for direct measurement of patient dose in X-ray diagnosis [20, 21]. To measure the absorbed dose in the lung, the OSLDs used were commercially available nanodots dosimeters (Landauer, Inc., Glenwood, USA). The nanodots OSLDs are 5 mm diameter, 0.2 mm thick disks infused with carbon-doped aluminum oxide (Al_2_O_3_:C). These discs are encased in a light-tight plastic case (10 × 10 × 2 mm) to prevent the depletion of optical signals due to light. The bar code information in the nanodots OSLDs was used for identification of each dosimeter and recording of the reading with ease. The MicroStar reader (Landauer Inc., Glenwood, USA) was used to read the optical signals of the OSLDs. The reader was calibrated, and the sensitivity of the dosimeter was corrected by the bar code including serial number and the sensitivity of each dosimeter. The reader system includes an external personal computer, installed with the InLight dose calculation software version 2.0.6.6 to acquire the data and export it to a Microsoft Excel spreadsheet for analysis. Additionally, before exposing nanodots OSLDs to X-rays, the nanodots OSLDs were prepared by annealing the residual signals to confirm no residual signals for radiation dose calculation remaining from earlier studies.

**Figure 1:**
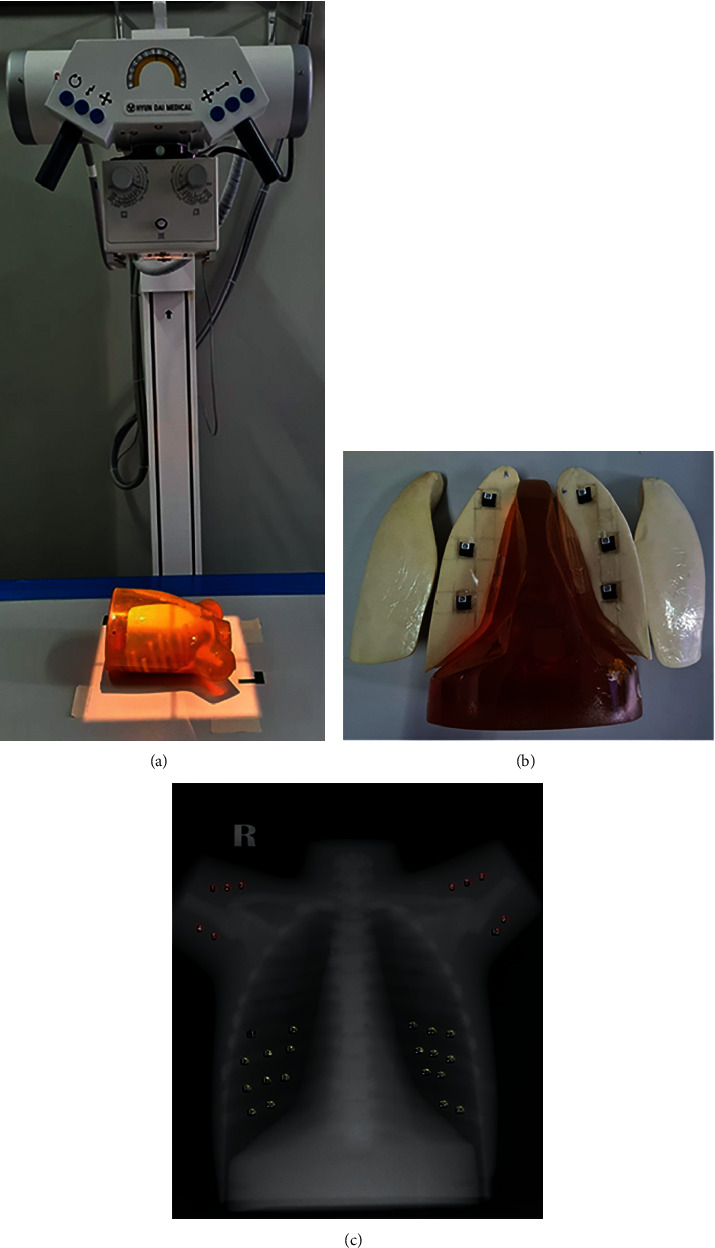
(a) The phantom settings for absorbed dose measurement; (b) the placing location of the nanodots OSLDs in the lung; (c) the location of the ROI measurements on the phantom image using the ImageJ software. The yellow circle and red circle represent the ROI object and ROI background, respectively.

The nanodots OSLDs were placed at the apex, middle, and base of both lungs in the phantom (Figure 1). The phantom was positioned on an X-ray table in the supine position by placing the apex of the lungs toward the anode side of the X-ray tube. The different exposure parameters were performed by altering kVp from 50 to 70 kV (5 kV increments) whilst altering the mAs to 1.6, 2, and 2.5. The pediatric chest phantom was exposed three times for each exposure. The reading of the nanodots OSLDs was recorded and the mean reading value for each exposure was reported.

### 2.4. Image Quality Evaluation

#### 2.4.1. Objective Image Quality: Measurements of SNR and CNR

Objective image quality was assessed by calculating the signal-to-noise ratio (SNR) and contrast-to-noise ratio (CNR). The ImageJ software [22] was used to evaluate the noise, SNR, and CNR in 15 radiographs. During the measurement process, all the ROIs must have the same size (100 mm^2^) and were placed at the same location for all the images. For noise measurements, the noise (standard deviation “*σ*”) was calculated using ImageJ software. SNR and CNR were calculated using the following [23]:

(1) $$\begin{array}{c} \text{SNR}=\frac{\text{average pixel value of object}}{\sigma_{\text{object}}}, \end{array}$$

(2) $$\begin{array}{c} \text{CNR }=\;\frac{\text{pixel value object}-\text{pixel value background}}{\sigma }. \end{array}$$

Furthermore, *σ* is calculated as $\sqrt{{\text{SD}}_{1}^{2}-{\text{SD}}_{2}^{2}/2}$, where SD_1_ is the standard deviation for the yellow ROI and SD_2_ is the standard deviation of the red ROI (Figure 1).

#### 2.4.2. Subjective Image Quality: Observer Study

Relative visual grading analysis was performed by two radiologists with at least five years of experience, interpreting radiographic images [24]. During the assessment, the observers were blinded to any information that could reveal how the images were acquired. In this study, the exposure technique of 65 kVp and 1.6 mAs was chosen as a reference image due to this technique being near the suitable exposure uses in pediatric chest supine (AP) in the DR and CR systems [25], and it is commonly used in pediatric clinical chest X-ray examination. To evaluate the image quality, the observers were invited to grade images based on the visualization of six criteria (Table 2) adapted from the European Guidelines on Quality Criteria for Diagnostic Radiographic Images [26]. Each review consisted of a pair of images displayed side-by-side of the reference image and the test image. The observer could select only one answer for each of the questions.

Image quality evaluation was performed using Diagnostic Medical Display JUSHA-M350 G (LCD monitors, MP, purchased from Thai GL Co., Ltd., Bangkok, Thailand) that were calibrated every year, together with the image workstations routinely used at the radiology department. The graphical user interface, INFINITT PACS (Thai GL Co., Ltd., Bangkok, Thailand), was used to display the images and record the radiologist's interpretation. The image evaluation process was conducted on a display (2,048 × 1,536) resolution monitor with a maximum brightness of 2,000 cd/m^2^.

### 2.5. Optimization: Figure of Merit

The figure of merit (FOM) was demonstrated and qualified the relationship between image quality and radiation dose. Therefore, it is applied to find the optimal exposure technique when considering both radiation dose and image quality.

The FOM was calculated to correlate the finding of absorbed organ dose and CNR. In this study, the absorbed organ dose, measured in mGy, was used as an indicator of dose. With CNR and absorbed organ dose, it was possible to calculate the figure-of-merit (FOM), which is described as follows [27]:

(3) $$\begin{array}{c} \text{the figure of merit}\left(\text{FOM}\right)=\frac{{\text{CNR}}^{2}}{\text{DOSE}}. \end{array}$$

### 2.6. Statistical Analysis

All results are presented as means ± standard deviation (SD). The data were compared using an ANOVA-like test. GraphPad Prism software (GraphPad, La Jolla, CA, USA) was employed for graphs and statistical analyses. Visual grading (ordinal variable) was also presented as median and range. A *P* value less than 0.05 was considered significant. The interobserver correlation was evaluated by the intraclass correlation coefficient (ICC) to assess the reliability of the observers, and the results were considered significant at the 95% confidence level by using SPSS Software Version 17.00 (SPSS, Inc., Chicago, IL, USA). The ICC value was interpreted as follows: poor (<0.5), moderate (0.5–0.75), good (0.75–0.9), and excellent (>0.9) reliability [28].

## 3. Results

### 3.1. Pediatric Chest Phantom

**Figure 2:**
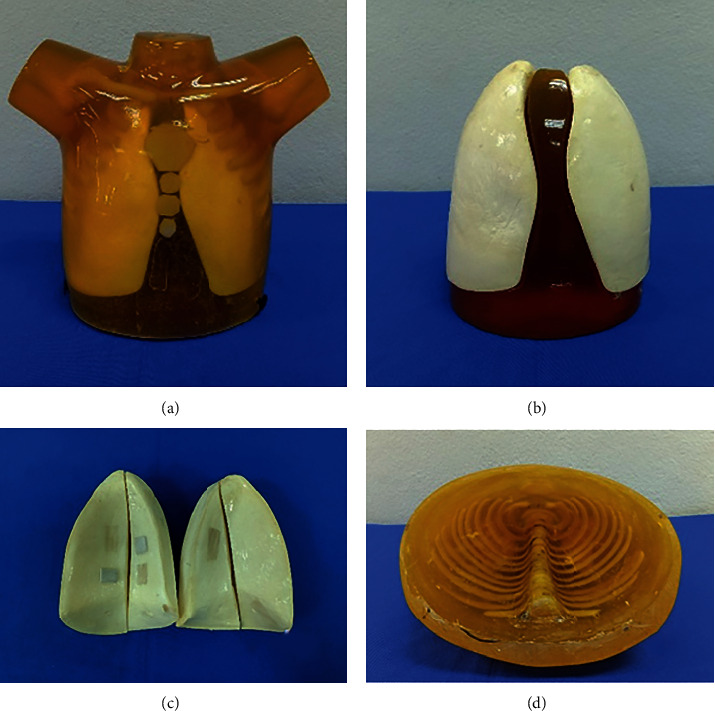
The constructed pediatric chest phantom: (a) the completed phantom; (b) the lung insertion part; (c) the left and right lungs; (d) the bone components (ribs and vertebra) in phantom.

The pediatric phantom was successfully fabricated as shown in Figure 2, which demonstrates the feasibility of using the developed and validation of tissue-equivalent materials to construct an anthropomorphic thorax phantom. The in-house mixtures for casting the model are carefully made, and it requires time to fill the entire phantom to avoid air bubbles and empty the model for lung insertion. The size of the obtained phantom was 18 cm × 22 cm with 7 cm of the thickness of the body, and this size is correlated with the standard body size of one-to-two-year-old children. For the lung insertion, the length of the lung, the width of the apex of both lungs, and the width of the base of both lungs were 14, 10, and 14 cm, respectively.

### 3.2. Estimation of Organ Absorbed Dose to the Lung

**Figure 3:**
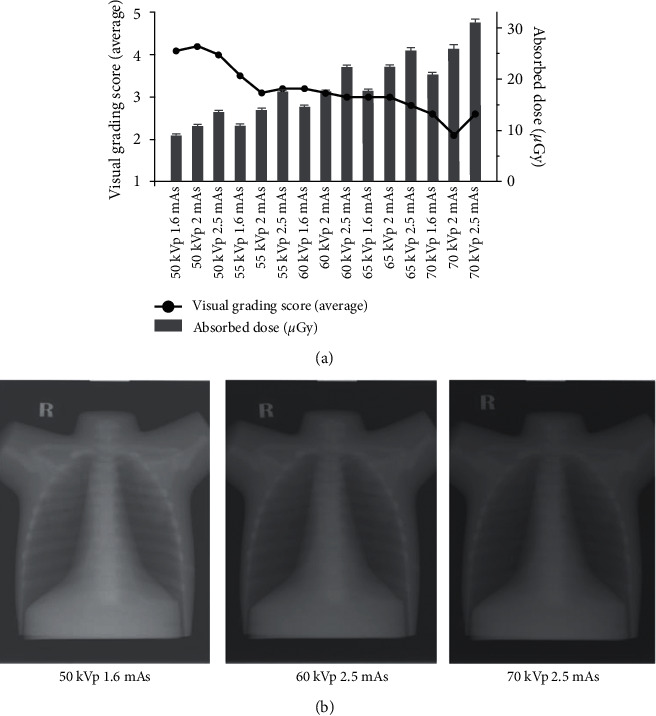
The resulting absorbed dose (mean ± SD) and the average visual grading analysis scoring (VGA) as a function of each exposure technique (a) Example of images in different image qualities obtained at different kV-mAs settings.

The average data of absorbed dose were obtained from the nanodots OSLDs reading at the apex, middle, and base of the left and right lung. No statistically significant difference in absorbed dose values between each placing location of the nanodots OSLDs was presented. Data on absorbed dose is summarized in Figure 3. It can be noted that when the exposure parameter (kVp and mAs) increased, the absorbed dose showed an increasing trend for all exposure settings. The highest absorbed dose, in this study, was approximately 0.031 mGy at 70 kVp and 2.5 mAs setting.

### 3.3. Objective Image Quality Evaluation

**Figure 4:**
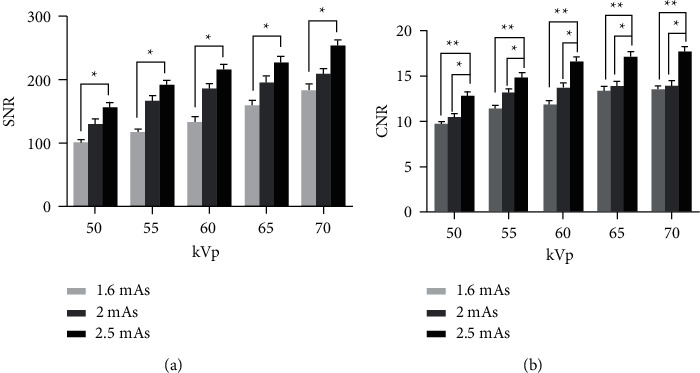
An increase in SNR (a) and CNR (b) as the exposure technique (kVp and mAs) increase. The asterisk indicates a significant difference (^*∗*^*p* < 0.05).

Figure 4 shows the result of SNR and CNR values with increasing variance in the kVp and mAs setting. Overall, using a higher kVp and mAs leads to higher SNR and CNR values. At the same kVp, the SNR value was gradually increased when mAs increased and had a statistically significant difference in each kVp at 2.5 mAs compared with 1.6 mAs.

Considering the CNR values, it shows an increasing trend across all kVp when mAs increase. Additionally, CNR is at 2.5 mAs in each kVp, and the value was statistically significant compared with 2 mAs and 1.6 mAs, respectively.

### 3.4. Subjective Image Quality Evaluation

Visual grading analysis scores (VGA) are also summarized in Figure 3. It can indicate that when the exposure parameters (kVp and mAs) increased, the visual grading scoring show a decreasing trend. The overall image quality was demonstrated as an equal quality compared with the reference image (average = 3.2 ± 0.3). The image that was acquired with 50 kVp and variation mAs was found to have a better image quality across all criteria compared with the reference image. In addition, to assess the interrater reliability of the observer, the ICC was obtained, and the result was 0.730 at a 95% CI (0.37–0.90; *p* < 0.05), indicating almost good reliability.

### 3.5. Overall Finding

**Figure 5:**
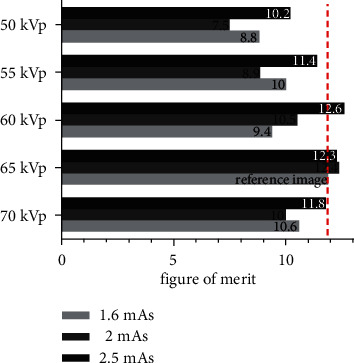
Result of the figure of merit.

The objective image quality (SNR/CNR) and the VGA were considered together with absorbed dose data results to identify the optimal exposure parameter (kVp and mAs) setting. The selection of the optimized protocol was based on it having no significant change in image quality from the reference standard protocol and having a reduction in absorbed dose. The summary of FOM was demonstrated in Figure 5. To consider the FOM data, the exposure technique setting at 60 kV and 2.5 mAs creates the best quality image according to the objective and subjective image quality and can be optimization radiation dose.

## 4. Discussion

This study focused on finding the exposure parameter setting (KVp and mAs) enabling optimization between image quality and dose in the variation of exposure technique for chest radiography in pediatric patients. The in-house developed pediatric chest phantom was used, which was designed and built using tissue-equivalent materials to represent a one-to-two-year-old child. The absorbed organ dose was estimated from the nanodots OSLDs measurement. Image quality was assessed using both objective and subjective assessments. The FOM shows the results of proper exposure technique settings for pediatric AP chest X-ray radiography.

Considering the in-house pediatric chest phantom, tissue-equivalent materials were chosen for constructing the phantom based on the data of density and the CT number validation. For constructing the phantom, polyester resin, polyester resin mixture with 40% CaCO3, and polyurethane foam were used to represent soft tissue, bone, and lung tissue, respectively. From Table 1, the density and CT number measurement of selected materials showed agreement between the tissue substitute used and human tissue for fabrication of the phantom. The percentage difference in CT number between soft-tissue and polyester resin was 41%. The percentage difference in soft tissue could affect the study results because the range of CT numbers for soft tissue should be between 40 and 80 HU. However, soft tissue can be comprised of various structures such as fat and muscle that have CT number values ranging from −190 to −30 HU for adipose tissue and −29 HU to 150 HU for muscle tissue [29]. Moreover, the polyester resin was used because it is an easily available and affordable material. Considering the bone tissue substitute (BTS), this study investigated the stability and CT density of the mixture between polyester resin and various concentrations of CaCO_3_. Polyester resin and 40% CaCO_3_ were chosen with good stability and have an acceptable range in CT numbers for bone (Table 1). Polyurethane foam was chosen as the lung tissue substitute (LTS) due to its use as a proposed lung substitute material for constructing the chest phantom described in previous studies [6]. The percentage difference in CT density between lung tissue and polyurethane foam was 59.3%, revealing a high difference. However, the CT number of polyurethane foam also matches the CT number of the inflated lung from the data of ICRU Report 44 [18]. Moreover, the CT number of lung tissue has been commonly measured by including air as well as the vessel in the lung, while the CT number of polyurethane foam represents the CT number for air only (approx., −1,000 HU).

Thus far, few studies have been carried out on the measurement of absorbed dose for chest X-ray examination in one-to-two-year-old children. Such studies will be challenging to investigate due to various factors such as novel detector technology as well as equipment. Previous research groups measured the absorbed organ dose on a beeswax-based pediatric phantom using the nanodots OSLDs. The results showed that the absorbed dose was increased when increasing the exposure with the reporting of absorbed dose in the lung at 0.047 mGy. Another researcher calculated the organ and effective doses by using the Monte Carlo PCXMC 2.0 code for estimation of the radiation risk from chest X-ray examination in pediatric patients. The results demonstrated that the organ doses for one-year-old patients in chest examinations were the lung with the value at 0.046 mGy [30]. Compared with the studies mentioned previously, our study showed similar results concerning increasing exposure techniques that can lead to increased radiation dose. However, certain factors may have affected the measurement of absorbed dose in this study. For instance, the phantom was made from tissue-equivalent material, so the density and X-ray attenuation properties are different from human tissue. From this point, the measurement of radiation dose can be obtained with an uncertainty value. Furthermore, our study decided to use the nanodots OSLDs for measuring the absorbed dose, though different types of dosimeter can be used for organ dose measurement, such as thermoluminescent dosimeters (TLDs) [31]. Further investigation should be conducted to compare the effect and result of each dosimetry used for absorbed dose measurement.

The exposure technique settings for pediatric chest radiography can vary significantly due to the discernible differences in patients' sizes. Consequently, different parameter settings may be necessary to obtain the best image results in similar anatomical regions among children of different ages. Moreover, pediatric radiography naturally has relatively low contrast. Thus, lowering the kVp setting increases image contrast, which could improve SNR and CNR and the tissue contrast [32, 33].

This study had some limitations. First, our in-house pediatric chest phantom is not fully representative of the pediatric thorax, indicating the absence of the lung parenchyma, lung vasculature, heart, and mediastinum. Second, it is acknowledged that a greater understanding of the impact of SID, filtration, or other parameters using our constructed phantom is warranted since the exposure technique settings were the only parameters investigated. Further studies should be performed.

## 5. Conclusion

In conclusion, our in-house developed phantom can be used successfully for estimation of absorbed organ dose and optimization of image quality for chest radiography in one-to-two-year-old pediatric patients. Furthermore, this study indicated that the recommended exposure technique for use during the pediatric chest AP examination is 60 kVp 2.5 mAs. Based on this exposure study, the use of nanodot OSLDs for organ absorbed dose measurement, SNR/CNR measurement, and visual grade scoring can help and offer significant information to optimize the trade-off between image quality and radiation dose.

## Figures and Tables

**Table 1:** The comparison of density and CT numbers of human tissue and tissue substitute materials. Polyester resin, polyester resin with 40% CaCO_3_, and polyurethane foam were used as soft tissue, bone, and lung, tissue-equivalent materials, respectively.

	Density (g/cm^3^)			CT number (HU)		
	Soft tissue	Bone	Lung	Soft tissue	Bone	Lung
Human tissue	1.06	1.6 to 2	0.26	40 to 80	400 to 1000	−400 to −600
Tissue substitutes used	1.12	n/a^*∗*^	0.30	112.8 ± 4.1	587.2 ± 51.1	−955.5 ± 1.4
% Difference	5.3	—	15.8	41	−41.3	59.3

^*∗*^n/a is not assessed.

**Table 2:** Criteria evaluation tool in the visual grading analysis for evaluation image.

#	Questions (criteria evaluation)					

1	Reproduction of the whole rib cage above the diaphragm.					
2	Visualization of the spine through the heart shadow.					
3	Comparing the sharpness of the right and left diaphragm between the image and the reference image.					
4	Comparing the contrast with the background for all the nodules between the image and the reference image.					
5	Less noise means better image quality; knowing this, what do you think of the image quality of this image?					
6	Comparing the differentiation between soft tissue, air, and bone on this image and the reference image.					

#	Image evaluation (Score)	Clearly inferior to the reference (−2)	Slightly inferior to the reference (−1)	Equal to the reference (0)	Slightly superior to the reference (1)	Clearly superior to the reference (2)

## Data Availability

The data that support the findings are available from the corresponding author upon reasonable request.
